# Maleopimaric acid acetic acid solvate

**DOI:** 10.1107/S1600536809023745

**Published:** 2009-06-27

**Authors:** Meng Zhang, Yong-hong Zhou, Xiao-xin Guo, Li-hong Hu

**Affiliations:** a Institute of Chemical Industry of Forest Products, Chinese Academy of Forestry, Nanjing, 210042, People’s Republic of China

## Abstract

The title compound, C_24_H_32_O_5_·C_2_H_4_O_2_, is a derivative of abietic acid. The two fused and unbridged cyclo­hexane rings have chair conformations and the anhydride ring is planar. Of the other three six-membered rings, two have boat conformations and one has a twist-boat conformation. The crystal structure is stabilized by inter­molecular O—H⋯O and C—H⋯O hydrogen bonds.

## Related literature

For general background, see: McCoy (2000 ▶); Schweizer *et al.* (2003 ▶); Savluchinske-Feio *et al.* (2007 ▶). For the crystal structure of a similar compound, see: Pan *et al.* (2006 ▶). For standard bond-length data, see: Allen *et al.* (1987 ▶).
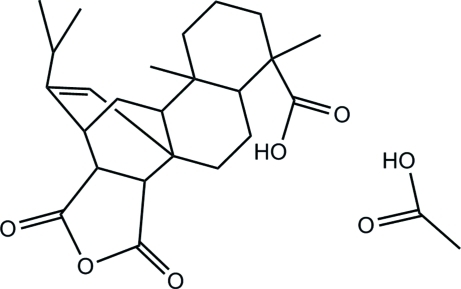


## Experimental

### 

#### Crystal data

C_24_H_32_O_5_·C_2_H_4_O_2_
*M* *_r_* = 460.55
Orthorhombic, 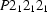

*a* = 7.9469 (10) Å
*b* = 12.7755 (16) Å
*c* = 24.884 (3) Å
*V* = 2526.3 (5) Å^3^
*Z* = 4
Mo *K*α radiation
μ = 0.09 mm^−1^
*T* = 291 K
0.30 × 0.26 × 0.24 mm

#### Data collection

Bruker SMART APEX CCD diffractometer
Absorption correction: multi-scan (*SADABS*; Bruker, 2000 ▶) *T*_min_ = 0.97, *T*_max_ = 0.98
13853 measured reflections
2837 independent reflections
2432 reflections with *I* > 2σ(*I*)
*R*_int_ = 0.059

#### Refinement

*R*[*F* ^2^ > 2σ(*F* ^2^)] = 0.047
*wR*(*F* ^2^) = 0.110
*S* = 1.04
2837 reflections
304 parameters
H-atom parameters constrained
Δρ_max_ = 0.19 e Å^−3^
Δρ_min_ = −0.17 e Å^−3^

### 

Data collection: *SMART* (Bruker, 2000 ▶); cell refinement: *SAINT* (Bruker, 2000 ▶); data reduction: *SAINT*; program(s) used to solve structure: *SHELXS97* (Sheldrick, 2008 ▶); program(s) used to refine structure: *SHELXL97* (Sheldrick, 2008 ▶); molecular graphics: *SHELXTL* (Sheldrick, 2008 ▶); software used to prepare material for publication: *SHELXTL*.

## Supplementary Material

Crystal structure: contains datablocks I, global. DOI: 10.1107/S1600536809023745/wn2334sup1.cif

Structure factors: contains datablocks I. DOI: 10.1107/S1600536809023745/wn2334Isup2.hkl

Additional supplementary materials:  crystallographic information; 3D view; checkCIF report

## Figures and Tables

**Table 1:** Hydrogen-bond geometry (Å, °)

*D*—H⋯*A*	*D*—H	H⋯*A*	*D*⋯*A*	*D*—H⋯*A*
O4—H4*C*⋯O6	0.93	1.70	2.617 (3)	169
O7—H7*A*⋯O5	0.93	1.76	2.681 (3)	171
C13—H13*C*⋯O5^i^	0.96	2.59	3.137 (5)	117
C26—H26*B*⋯O1^ii^	0.96	2.56	3.369 (4)	142

Symmetry codes: (i) 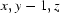; (ii) 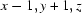.

**Fig. 1.**
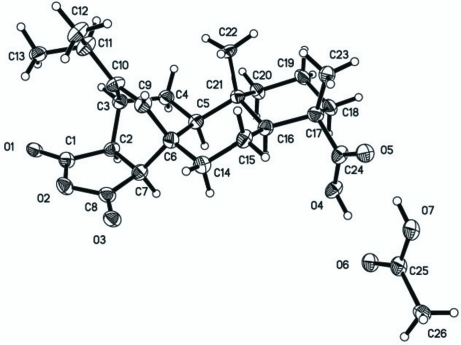
A view of the molecular structure of the title compound, showing displacement ellipsoids at the 30% probability level. Hydrogen atoms are drawn as spheres of arbitrary radius.

**Fig. 2.**
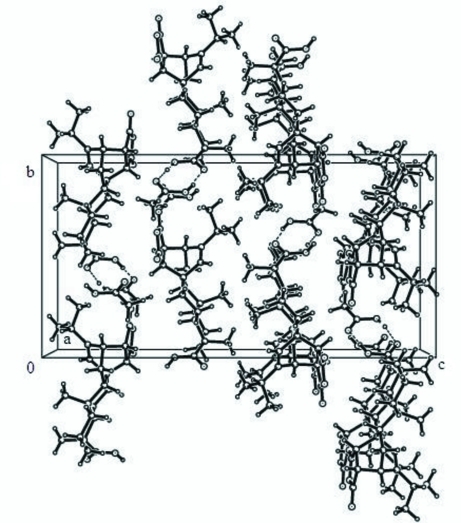
The packing of the title compound, viewed along the a axis. Dashed lines indicate hydrogen bonds.

## supplementary crystallographic information

### Comment

Rosin, a versatile natural resin, possesses a rare combination of many desirable properties and has consequently found innumerable industrial uses in a modified form or in conjunction with other natural or synthetic resins (McCoy, 2000). Abietic type resin acid is the major component of gum rosin, including abietic acid, neoabietic acid, levo-pimaric acid, palustric acid and is a high quality biomass resource in developing chiral new drugs (Schweizer *et al.*, 2003). Abietic acid and its derivatives are readily available hydrophenanthrene compounds which form useful starting materials for the design and synthesis of industrially and physiologically important products (Savluchinske-Feio *et al.*,2007).

The crystal structure of a similar compound, also a derivative of maleopimaric acid, has already been published (Pan *et al.*, 2006). The molecular structure of the title compound, is shown in Fig. 1. The asymmetric unit consists of two molecules, viz. maleopimaric anhydride and acetic acid. The cyclohexane rings C5, C6, C14–C16, C21 and C16—C21 have typical chair forms. The cyclohexane ring C2–C7 has a slightly distorted twist-boat conformation; the other two six-membered rings adopt boat conformations. The configuration about the C9═C10 bond is *Z* (Fig. 1), with the H atom and the isopropyl group *cis* with respect to each other. The bond lengths (Allen *et al.*, 1987) and bond angles exhibit normal values. In the crystal structure, the molecules are linked (Fig.2) by O—H···O and C—H···O intermolecular hydrogen bonds, also by van der Waals forces.

### Experimental

Rosin (10.0 g), acetic acid (7 ml), and maleic anhydride (3.0 g) were put into a 50-ml three-necked flask and magnetically stirred; the mixture was stirred for 20 min with power 450w. The solution was put into 5 ml glacial acetic acid and cooled, washed with hot water (10 ml), dried (MgSO_4_), and concentrated to dryness. Recrystallization from ethanol afforded the adduct (7.5 g, 50%).

### Refinement

All H atoms were initially located in a difference Fourier map. The methyl H atoms were then constrained to an ideal geometry, with C—H distances of 0.96 Å and *U*_iso_(H) = 1.5*U*_eq_, but each group was allowed to rotate freely about its C—C bond. All other H atoms were placed in geometrically idealized positions and constrained to ride on their parent atoms,with C—H distances in the range 0.93–0.98 Å and *U*_iso_(H) = 1.2*U*_eq_(C); O—H = 0.93 Å and *U*_iso_(H) = 1.2*U*_eq_(O). In the absence of significant anomalous scattering effects, Friedel pairs were merged.

### Crystal data

**Table:**

C_24_H_32_O_5_·C_2_H_4_O_2_	*D*_x_ = 1.211 Mg m^−^^3^
*M**_r_* = 460.55	Melting point: 498 K
Orthorhombic, *P*2_1_2_1_2_1_	Mo *K*α radiation, λ = 0.71073 Å
Hall symbol: P 2ac 2ab	Cell parameters from 3636 reflections
*a* = 7.9469 (10) Å	θ = 2.3–23.2°
*b* = 12.7755 (16) Å	µ = 0.09 mm^−^^1^
*c* = 24.884 (3) Å	*T* = 291 K
*V* = 2526.3 (5) Å^3^	Block, colorless
*Z* = 4	0.30 × 0.26 × 0.24 mm
*F*(000) = 992	

### Data collection

**Table:**

Bruker SMART APEX CCD diffractometer	2837 independent reflections
Radiation source: sealed tube	2432 reflections with *I* > 2σ(*I*)
graphite	*R*_int_ = 0.059
φ and ω scans	θ_max_ = 26.0°, θ_min_ = 1.8°
Absorption correction: multi-scan (*SADABS*; Bruker, 2000)	*h* = −9→8
*T*_min_ = 0.97, *T*_max_ = 0.98	*k* = −15→15
13853 measured reflections	*l* = −27→30

### Refinement

**Table:**

Refinement on *F*^2^	Secondary atom site location: difference Fourier map
Least-squares matrix: full	Hydrogen site location: inferred from neighbouring sites
*R*[*F*^2^ > 2σ(*F*^2^)] = 0.047	H-atom parameters constrained
*wR*(*F*^2^) = 0.110	*w* = 1/[σ^2^(*F*_o_^2^) + (0.05*P*)^2^ + 0.55*P*] where *P* = (*F*_o_^2^ + 2*F*_c_^2^)/3
*S* = 1.04	(Δ/σ)_max_ < 0.001
2837 reflections	Δρ_max_ = 0.19 e Å^−^^3^
304 parameters	Δρ_min_ = −0.17 e Å^−^^3^
0 restraints	Extinction correction: *SHELXL97* (Sheldrick, 2008), Fc^*^=kFc[1+0.001xFc^2^λ^3^/sin(2θ)]^-1/4^
Primary atom site location: structure-invariant direct methods	Extinction coefficient: 0.0076 (9)

### Special details

**Table:**

Geometry. All e.s.d.'s (except the e.s.d. in the dihedral angle between two l.s. planes) are estimated using the full covariance matrix. The cell e.s.d.'s are taken into account individually in the estimation of e.s.d.'s in distances, angles and torsion angles; correlations between e.s.d.'s in cell parameters are only used when they are defined by crystal symmetry. An approximate (isotropic) treatment of cell e.s.d.'s is used for estimating e.s.d.'s involving l.s. planes.
Refinement. Refinement of *F*^2^ against ALL reflections. The weighted *R*-factor *wR* and goodness of fit *S* are based on *F*^2^, conventional *R*-factors *R* are based on *F*, with *F* set to zero for negative *F*^2^. The threshold expression of *F*^2^ > σ(*F*^2^) is used only for calculating *R*-factors(gt) *etc*. and is not relevant to the choice of reflections for refinement. *R*-factors based on *F*^2^ are statistically about twice as large as those based on *F*, and *R*- factors based on ALL data will be even larger. The Friedel pairs have been merged in the absence of anomalous scattering.

### Fractional atomic coordinates and isotropic or equivalent isotropic displacement parameters (Å^2^)

**Table:**

	*x*	*y*	*z*	*U*_iso_*/*U*_eq_	
C1	0.5002 (4)	−0.1408 (3)	0.21013 (13)	0.0419 (7)	
C2	0.5980 (4)	−0.0411 (2)	0.20070 (12)	0.0398 (7)	
H2	0.6794	−0.0301	0.2297	0.048*	
C3	0.6887 (4)	−0.0420 (2)	0.14559 (13)	0.0417 (7)	
H3	0.7696	−0.0998	0.1434	0.050*	
C4	0.7802 (4)	0.0670 (2)	0.14272 (13)	0.0426 (7)	
H4A	0.8616	0.0723	0.1716	0.051*	
H4B	0.8400	0.0730	0.1089	0.051*	
C5	0.6514 (4)	0.1559 (2)	0.14754 (14)	0.0411 (7)	
H5	0.6683	0.1867	0.1832	0.049*	
C6	0.4713 (4)	0.1086 (2)	0.14794 (13)	0.0414 (7)	
C7	0.4657 (4)	0.0448 (2)	0.20125 (12)	0.0409 (7)	
H7	0.4851	0.0916	0.2318	0.049*	
C8	0.3020 (4)	−0.0147 (3)	0.20917 (13)	0.0442 (8)	
C9	0.4523 (4)	0.0321 (3)	0.10233 (12)	0.0429 (7)	
H9	0.3687	0.0390	0.0764	0.052*	
C10	0.5648 (4)	−0.0472 (3)	0.10145 (12)	0.0453 (8)	
C11	0.5732 (5)	−0.1285 (3)	0.05890 (15)	0.0552 (10)	
H11	0.6773	−0.1203	0.0383	0.066*	
C12	0.4230 (5)	−0.1334 (3)	0.02054 (15)	0.0553 (10)	
H12A	0.3219	−0.1455	0.0407	0.083*	
H12B	0.4391	−0.1895	−0.0046	0.083*	
H12C	0.4137	−0.0683	0.0015	0.083*	
C13	0.5604 (5)	−0.2392 (3)	0.07953 (13)	0.0503 (9)	
H13A	0.6437	−0.2503	0.1069	0.075*	
H13B	0.5788	−0.2873	0.0505	0.075*	
H13C	0.4504	−0.2505	0.0944	0.075*	
C14	0.3351 (4)	0.1953 (2)	0.14874 (13)	0.0434 (7)	
H14A	0.2267	0.1642	0.1406	0.052*	
H14B	0.3287	0.2247	0.1846	0.052*	
C15	0.3695 (4)	0.2827 (2)	0.10897 (13)	0.0427 (8)	
H15A	0.3630	0.2555	0.0726	0.051*	
H15B	0.2849	0.3369	0.1128	0.051*	
C16	0.5419 (4)	0.3288 (2)	0.11854 (13)	0.0409 (7)	
H16	0.5485	0.3405	0.1574	0.049*	
C17	0.5691 (4)	0.4405 (3)	0.09213 (12)	0.0408 (7)	
C18	0.7484 (4)	0.4797 (3)	0.10755 (14)	0.0442 (8)	
H18A	0.7707	0.5453	0.0893	0.053*	
H18B	0.7528	0.4925	0.1459	0.053*	
C19	0.8818 (4)	0.4017 (3)	0.09266 (15)	0.0491 (9)	
H19A	0.9912	0.4293	0.1026	0.059*	
H19B	0.8809	0.3914	0.0540	0.059*	
C20	0.8550 (4)	0.2952 (3)	0.12078 (14)	0.0450 (8)	
H20A	0.8616	0.3047	0.1594	0.054*	
H20B	0.9437	0.2474	0.1102	0.054*	
C21	0.6810 (4)	0.2471 (2)	0.10598 (12)	0.0405 (7)	
C22	0.6830 (4)	0.2061 (3)	0.04687 (12)	0.0429 (7)	
H22A	0.5701	0.1906	0.0357	0.064*	
H22B	0.7501	0.1437	0.0449	0.064*	
H22C	0.7300	0.2587	0.0238	0.064*	
C23	0.5378 (5)	0.4443 (3)	0.03186 (12)	0.0445 (7)	
H23A	0.4192	0.4508	0.0252	0.067*	
H23B	0.5789	0.3812	0.0156	0.067*	
H23C	0.5955	0.5034	0.0168	0.067*	
C24	0.4419 (4)	0.5116 (3)	0.11930 (13)	0.0426 (7)	
C25	0.1024 (4)	0.6778 (3)	0.18987 (14)	0.0460 (8)	
C26	−0.0272 (4)	0.7410 (3)	0.21888 (13)	0.0484 (8)	
H26A	−0.0330	0.7187	0.2557	0.073*	
H26B	−0.1348	0.7313	0.2021	0.073*	
H26C	0.0031	0.8137	0.2175	0.073*	
O1	0.5518 (3)	−0.22935 (16)	0.21417 (9)	0.0463 (6)	
O2	0.3332 (3)	−0.12062 (18)	0.21447 (10)	0.0472 (6)	
O3	0.1594 (3)	0.01506 (17)	0.21128 (9)	0.0453 (5)	
O4	0.4493 (3)	0.51827 (17)	0.17031 (8)	0.0455 (6)	
H4C	0.3728	0.5602	0.1887	0.055*	
O5	0.3379 (3)	0.55953 (19)	0.09249 (8)	0.0472 (6)	
O6	0.2043 (3)	0.62457 (17)	0.21453 (9)	0.0465 (6)	
O7	0.1034 (3)	0.68154 (18)	0.13804 (9)	0.0462 (6)	
H7A	0.1831	0.6430	0.1191	0.055*	

### Atomic displacement parameters (Å^2^)

**Table:**

	*U*^11^	*U*^22^	*U*^33^	*U*^12^	*U*^13^	*U*^23^
C1	0.0453 (18)	0.0411 (16)	0.0395 (17)	0.0089 (14)	−0.0018 (14)	0.0098 (14)
C2	0.0398 (17)	0.0409 (17)	0.0389 (16)	0.0087 (14)	0.0007 (13)	−0.0023 (13)
C3	0.0443 (17)	0.0377 (15)	0.0431 (17)	0.0077 (14)	0.0041 (15)	0.0005 (13)
C4	0.0427 (17)	0.0423 (17)	0.0428 (17)	0.0049 (14)	0.0031 (14)	0.0012 (14)
C5	0.0417 (17)	0.0386 (16)	0.0430 (17)	0.0036 (13)	0.0036 (15)	−0.0003 (13)
C6	0.0400 (16)	0.0405 (16)	0.0437 (17)	0.0004 (13)	−0.0006 (15)	−0.0001 (14)
C7	0.0403 (17)	0.0403 (16)	0.0420 (17)	0.0049 (14)	−0.0029 (14)	−0.0019 (13)
C8	0.0434 (18)	0.0452 (18)	0.0441 (18)	0.0096 (15)	0.0095 (15)	0.0081 (14)
C9	0.0470 (17)	0.0430 (17)	0.0388 (16)	0.0055 (15)	−0.0113 (15)	−0.0024 (13)
C10	0.0439 (18)	0.0488 (19)	0.0433 (17)	0.0118 (15)	0.0043 (15)	−0.0102 (14)
C11	0.067 (2)	0.0469 (19)	0.051 (2)	0.0168 (19)	−0.0152 (19)	−0.0133 (16)
C12	0.059 (2)	0.050 (2)	0.057 (2)	0.0163 (18)	−0.0149 (19)	−0.0216 (17)
C13	0.0488 (19)	0.056 (2)	0.0461 (19)	−0.0147 (17)	0.0107 (16)	−0.0149 (15)
C14	0.0414 (17)	0.0470 (17)	0.0418 (17)	0.0044 (15)	−0.0055 (15)	0.0019 (14)
C15	0.0506 (19)	0.0365 (16)	0.0411 (17)	0.0070 (15)	−0.0127 (15)	−0.0028 (13)
C16	0.0417 (17)	0.0392 (16)	0.0418 (17)	0.0102 (14)	0.0007 (14)	0.0027 (13)
C17	0.0387 (16)	0.0441 (17)	0.0397 (15)	0.0031 (14)	0.0028 (14)	0.0037 (14)
C18	0.0451 (17)	0.0406 (18)	0.0469 (18)	−0.0079 (15)	0.0056 (15)	0.0080 (14)
C19	0.0444 (18)	0.051 (2)	0.0522 (19)	−0.0018 (15)	0.0170 (16)	0.0135 (16)
C20	0.0445 (18)	0.0445 (18)	0.0459 (18)	−0.0015 (15)	0.0029 (15)	0.0114 (15)
C21	0.0416 (17)	0.0394 (16)	0.0406 (16)	0.0058 (14)	0.0125 (14)	0.0013 (13)
C22	0.0426 (17)	0.0441 (17)	0.0420 (17)	0.0118 (15)	0.0126 (14)	−0.0080 (14)
C23	0.0463 (18)	0.0417 (17)	0.0456 (17)	0.0112 (15)	0.0012 (15)	0.0056 (14)
C24	0.0422 (17)	0.0411 (17)	0.0443 (17)	0.0118 (15)	−0.0131 (14)	−0.0023 (13)
C25	0.0419 (18)	0.0466 (18)	0.0496 (19)	0.0143 (15)	−0.0064 (15)	0.0150 (15)
C26	0.0450 (18)	0.0536 (19)	0.0468 (18)	0.0114 (16)	0.0139 (16)	0.0172 (16)
O1	0.0498 (13)	0.0413 (12)	0.0480 (12)	0.0178 (11)	0.0177 (12)	0.0161 (10)
O2	0.0437 (13)	0.0468 (12)	0.0511 (13)	0.0058 (11)	0.0054 (12)	0.0177 (10)
O3	0.0417 (13)	0.0465 (12)	0.0477 (12)	0.0104 (11)	0.0100 (11)	0.0107 (10)
O4	0.0480 (13)	0.0462 (12)	0.0422 (12)	0.0174 (11)	−0.0129 (11)	−0.0074 (9)
O5	0.0502 (13)	0.0539 (14)	0.0376 (11)	0.0186 (11)	−0.0092 (11)	0.0108 (10)
O6	0.0465 (13)	0.0495 (13)	0.0436 (12)	0.0148 (11)	−0.0067 (11)	0.0144 (10)
O7	0.0442 (13)	0.0469 (12)	0.0475 (13)	0.0169 (10)	−0.0119 (10)	0.0153 (11)

### Geometric parameters (Å, °)

**Table:**

C1—O1	1.207 (4)	C14—H14B	0.9700
C1—O2	1.356 (4)	C15—C16	1.510 (5)
C1—C2	1.511 (5)	C15—H15A	0.9700
C2—C7	1.520 (4)	C15—H15B	0.9700
C2—C3	1.550 (4)	C16—C21	1.552 (4)
C2—H2	0.9800	C16—C17	1.586 (4)
C3—C10	1.477 (5)	C16—H16	0.9800
C3—C4	1.573 (4)	C17—C24	1.518 (5)
C3—H3	0.9800	C17—C23	1.521 (4)
C4—C5	1.533 (4)	C17—C18	1.558 (5)
C4—H4A	0.9700	C18—C19	1.501 (5)
C4—H4B	0.9700	C18—H18A	0.9700
C5—C6	1.553 (4)	C18—H18B	0.9700
C5—C21	1.576 (4)	C19—C20	1.544 (4)
C5—H5	0.9800	C19—H19A	0.9700
C6—C9	1.505 (4)	C19—H19B	0.9700
C6—C14	1.549 (4)	C20—C21	1.557 (5)
C6—C7	1.557 (4)	C20—H20A	0.9700
C7—C8	1.519 (5)	C20—H20B	0.9700
C7—H7	0.9800	C21—C22	1.561 (4)
C8—O3	1.197 (4)	C22—H22A	0.9600
C8—O2	1.382 (4)	C22—H22B	0.9600
C9—C10	1.351 (5)	C22—H22C	0.9600
C9—H9	0.9300	C23—H23A	0.9600
C10—C11	1.485 (4)	C23—H23B	0.9600
C11—C13	1.507 (5)	C23—H23C	0.9600
C11—C12	1.529 (5)	C24—O5	1.226 (4)
C11—H11	0.9800	C24—O4	1.274 (4)
C12—H12A	0.9600	C25—O6	1.223 (4)
C12—H12B	0.9600	C25—O7	1.291 (4)
C12—H12C	0.9600	C25—C26	1.494 (5)
C13—H13A	0.9600	C26—H26A	0.9600
C13—H13B	0.9600	C26—H26B	0.9600
C13—H13C	0.9600	C26—H26C	0.9600
C14—C15	1.517 (4)	O4—H4C	0.9300
C14—H14A	0.9700	O7—H7A	0.9300
			
O1—C1—O2	120.3 (3)	H14A—C14—H14B	107.8
O1—C1—C2	128.9 (3)	C16—C15—C14	110.3 (3)
O2—C1—C2	110.8 (3)	C16—C15—H15A	109.6
C1—C2—C7	104.6 (3)	C14—C15—H15A	109.6
C1—C2—C3	111.8 (3)	C16—C15—H15B	109.6
C7—C2—C3	109.6 (2)	C14—C15—H15B	109.6
C1—C2—H2	110.3	H15A—C15—H15B	108.1
C7—C2—H2	110.3	C15—C16—C21	110.7 (3)
C3—C2—H2	110.3	C15—C16—C17	114.1 (3)
C10—C3—C2	110.4 (3)	C21—C16—C17	115.1 (3)
C10—C3—C4	108.3 (3)	C15—C16—H16	105.3
C2—C3—C4	104.4 (3)	C21—C16—H16	105.3
C10—C3—H3	111.2	C17—C16—H16	105.3
C2—C3—H3	111.2	C24—C17—C23	108.1 (3)
C4—C3—H3	111.2	C24—C17—C18	107.9 (3)
C5—C4—C3	110.1 (3)	C23—C17—C18	112.5 (3)
C5—C4—H4A	109.6	C24—C17—C16	105.3 (3)
C3—C4—H4A	109.6	C23—C17—C16	114.5 (3)
C5—C4—H4B	109.6	C18—C17—C16	108.2 (3)
C3—C4—H4B	109.6	C19—C18—C17	111.8 (3)
H4A—C4—H4B	108.2	C19—C18—H18A	109.2
C4—C5—C6	109.1 (2)	C17—C18—H18A	109.2
C4—C5—C21	113.4 (3)	C19—C18—H18B	109.2
C6—C5—C21	115.4 (3)	C17—C18—H18B	109.2
C4—C5—H5	106.1	H18A—C18—H18B	107.9
C6—C5—H5	106.1	C18—C19—C20	112.0 (3)
C21—C5—H5	106.1	C18—C19—H19A	109.2
C9—C6—C14	113.8 (3)	C20—C19—H19A	109.2
C9—C6—C5	109.9 (3)	C18—C19—H19B	109.2
C14—C6—C5	111.5 (2)	C20—C19—H19B	109.2
C9—C6—C7	107.5 (3)	H19A—C19—H19B	107.9
C14—C6—C7	110.1 (3)	C19—C20—C21	111.3 (3)
C5—C6—C7	103.6 (3)	C19—C20—H20A	109.4
C8—C7—C2	103.4 (2)	C21—C20—H20A	109.4
C8—C7—C6	113.4 (3)	C19—C20—H20B	109.4
C2—C7—C6	110.5 (3)	C21—C20—H20B	109.4
C8—C7—H7	109.8	H20A—C20—H20B	108.0
C2—C7—H7	109.8	C16—C21—C20	108.6 (3)
C6—C7—H7	109.8	C16—C21—C22	115.0 (3)
O3—C8—O2	118.4 (3)	C20—C21—C22	110.3 (3)
O3—C8—C7	131.1 (3)	C16—C21—C5	105.0 (2)
O2—C8—C7	110.4 (3)	C20—C21—C5	105.6 (3)
C10—C9—C6	115.6 (3)	C22—C21—C5	111.8 (3)
C10—C9—H9	122.2	C21—C22—H22A	109.5
C6—C9—H9	122.2	C21—C22—H22B	109.5
C9—C10—C3	113.3 (3)	H22A—C22—H22B	109.5
C9—C10—C11	124.5 (3)	C21—C22—H22C	109.5
C3—C10—C11	122.1 (3)	H22A—C22—H22C	109.5
C10—C11—C13	114.2 (3)	H22B—C22—H22C	109.5
C10—C11—C12	116.0 (3)	C17—C23—H23A	109.5
C13—C11—C12	97.0 (3)	C17—C23—H23B	109.5
C10—C11—H11	109.7	H23A—C23—H23B	109.5
C13—C11—H11	109.7	C17—C23—H23C	109.5
C12—C11—H11	109.7	H23A—C23—H23C	109.5
C11—C12—H12A	109.5	H23B—C23—H23C	109.5
C11—C12—H12B	109.5	O5—C24—O4	122.7 (3)
H12A—C12—H12B	109.5	O5—C24—C17	120.4 (3)
C11—C12—H12C	109.5	O4—C24—C17	116.9 (3)
H12A—C12—H12C	109.5	O6—C25—O7	121.2 (3)
H12B—C12—H12C	109.5	O6—C25—C26	121.0 (3)
C11—C13—H13A	109.5	O7—C25—C26	117.9 (3)
C11—C13—H13B	109.5	C25—C26—H26A	109.5
H13A—C13—H13B	109.5	C25—C26—H26B	109.5
C11—C13—H13C	109.5	H26A—C26—H26B	109.5
H13A—C13—H13C	109.5	C25—C26—H26C	109.5
H13B—C13—H13C	109.5	H26A—C26—H26C	109.5
C15—C14—C6	113.1 (3)	H26B—C26—H26C	109.5
C15—C14—H14A	109.0	C1—O2—C8	110.7 (3)
C6—C14—H14A	109.0	C24—O4—H4C	119.9
C15—C14—H14B	109.0	C25—O7—H7A	119.5
C6—C14—H14B	109.0		
			
O1—C1—C2—C7	−178.2 (3)	C3—C10—C11—C12	171.8 (3)
O2—C1—C2—C7	1.4 (4)	C9—C6—C14—C15	−79.4 (3)
O1—C1—C2—C3	63.3 (5)	C5—C6—C14—C15	45.5 (4)
O2—C1—C2—C3	−117.1 (3)	C7—C6—C14—C15	159.9 (3)
C1—C2—C3—C10	63.1 (3)	C6—C14—C15—C16	−55.4 (4)
C7—C2—C3—C10	−52.4 (3)	C14—C15—C16—C21	66.0 (3)
C1—C2—C3—C4	179.3 (3)	C14—C15—C16—C17	−162.3 (3)
C7—C2—C3—C4	63.8 (3)	C15—C16—C17—C24	62.4 (3)
C10—C3—C4—C5	58.8 (3)	C21—C16—C17—C24	−168.1 (3)
C2—C3—C4—C5	−58.8 (3)	C15—C16—C17—C23	−56.2 (4)
C3—C4—C5—C6	−5.6 (4)	C21—C16—C17—C23	73.3 (4)
C3—C4—C5—C21	−135.8 (3)	C15—C16—C17—C18	177.5 (3)
C4—C5—C6—C9	−48.9 (3)	C21—C16—C17—C18	−53.0 (3)
C21—C5—C6—C9	80.1 (3)	C24—C17—C18—C19	167.7 (3)
C4—C5—C6—C14	−176.0 (3)	C23—C17—C18—C19	−73.2 (4)
C21—C5—C6—C14	−47.0 (4)	C16—C17—C18—C19	54.3 (3)
C4—C5—C6—C7	65.7 (3)	C17—C18—C19—C20	−59.5 (4)
C21—C5—C6—C7	−165.3 (3)	C18—C19—C20—C21	59.2 (4)
C1—C2—C7—C8	−1.8 (3)	C15—C16—C21—C20	−175.5 (3)
C3—C2—C7—C8	118.1 (3)	C17—C16—C21—C20	53.2 (3)
C1—C2—C7—C6	−123.5 (3)	C15—C16—C21—C22	60.3 (4)
C3—C2—C7—C6	−3.5 (4)	C17—C16—C21—C22	−70.9 (4)
C9—C6—C7—C8	−60.5 (3)	C15—C16—C21—C5	−62.9 (3)
C14—C6—C7—C8	64.0 (3)	C17—C16—C21—C5	165.8 (3)
C5—C6—C7—C8	−176.7 (2)	C19—C20—C21—C16	−54.0 (3)
C9—C6—C7—C2	55.1 (3)	C19—C20—C21—C22	72.8 (3)
C14—C6—C7—C2	179.5 (3)	C19—C20—C21—C5	−166.2 (3)
C5—C6—C7—C2	−61.2 (3)	C4—C5—C21—C16	−178.6 (3)
C2—C7—C8—O3	−178.1 (4)	C6—C5—C21—C16	54.4 (3)
C6—C7—C8—O3	−58.4 (5)	C4—C5—C21—C20	−63.9 (3)
C2—C7—C8—O2	1.8 (3)	C6—C5—C21—C20	169.2 (3)
C6—C7—C8—O2	121.5 (3)	C4—C5—C21—C22	56.1 (4)
C14—C6—C9—C10	−177.5 (3)	C6—C5—C21—C22	−70.9 (3)
C5—C6—C9—C10	56.7 (4)	C23—C17—C24—O5	0.2 (4)
C7—C6—C9—C10	−55.3 (4)	C18—C17—C24—O5	122.0 (3)
C6—C9—C10—C3	−1.2 (4)	C16—C17—C24—O5	−122.7 (3)
C6—C9—C10—C11	−177.3 (3)	C23—C17—C24—O4	−180.0 (3)
C2—C3—C10—C9	57.3 (4)	C18—C17—C24—O4	−58.1 (4)
C4—C3—C10—C9	−56.4 (4)	C16—C17—C24—O4	57.2 (4)
C2—C3—C10—C11	−126.5 (3)	O1—C1—O2—C8	179.4 (3)
C4—C3—C10—C11	119.8 (4)	C2—C1—O2—C8	−0.2 (4)
C9—C10—C11—C13	−124.0 (4)	O3—C8—O2—C1	178.9 (3)
C3—C10—C11—C13	60.1 (5)	C7—C8—O2—C1	−1.1 (4)
C9—C10—C11—C12	−12.4 (6)		

### Hydrogen-bond geometry (Å, °)

**Table:**

*D*—H···*A*	*D*—H	H···*A*	*D*···*A*	*D*—H···*A*
O4—H4C···O6	0.93	1.70	2.617 (3)	169
O7—H7A···O5	0.93	1.76	2.681 (3)	171
C13—H13C···O5^i^	0.96	2.59	3.137 (5)	117
C26—H26B···O1^ii^	0.96	2.56	3.369 (4)	142

Symmetry codes: (i) *x*, *y*−1, *z*; (ii) *x*−1, *y*+1, *z*.
